# Smoking decreases the level of circulating CD34+ progenitor cells in young healthy women - a pilot study

**DOI:** 10.1186/1472-6874-10-20

**Published:** 2010-05-30

**Authors:** Antje Ludwig, Nicoline Jochmann, Andras Kertesz, Claudia Kuhn, Simone Mueller, Christine Gericke, Gert Baumann, Karl Stangl, Verena Stangl

**Affiliations:** 1 Med. Klinik mit Schwerpunkt Kardiologie und Angiologie, Charité - Universitaetsmedizin Berlin, Campus Mitte, Berlin, Germany; 2 Institut für Biometrie und Klinische Epidemiologie, Charité - Universitaetsmedizin Berlin, Campus Mitte, Berlin, Germany

## Abstract

**Background:**

Decreased levels of circulating bone marrow-derived progenitor cells have been associated with risk factors and cardiovascular diseases. Smoking is the most important modifiable risk factor for atherosclerosis in young women. The aim of this pilot study was to assess in healthy premenopausal women without other risk factors for cardiovascular disease the influence of nicotine abuse on the number of circulating progenitor cells in relation to endothelial function.

**Methods:**

The number of endothelial progenitor cells, measured as colony-forming units in a cell-culture assay (EPC-CFU) and the number of circulating CD34 + and CD34 + /CD133 + cells, measured by flow cytometry, was estimated in 32 women at the menstrual phase of the menstrual cycle. In addition, flow-mediated dilation (FMD) was assessed as a marker for vascular function. In a subgroup of these women (n = 20), progenitor cells were also investigated at the mid-follicular and luteal phases of the menstrual cycle.

**Results:**

Compared to non-smokers, the abundance of circulating CD34 + cells was significantly lower in smoking women in the menstrual, mid-luteal, and mid-follicular phases of the menstrual cycle. The number of CD34 + progenitor cells was revealed to have significant positive correlation with FMD in young healthy women, whereas CD34 + /CD133 + progenitor cells and EPC-CFU showed no significant correlation.

**Conclusion:**

The number of CD34 + progenitor cells positively correlates with FMD in young healthy women and is decreased by smoking.

## Background

Vascular homeostasis is controlled not solely by cells of the vessel wall, but also by circulating bone marrow-derived progenitor cells (PCs). In particular, a subset of circulating stem cells, designated endothelial progenitor cells (EPCs), is considered to contribute to endothelial cell regeneration and neovascularisation [1]. Levels of circulating PCs/EPCs have been correlated with endothelial function, atherogenic risk factors, as well as various cardiovascular diseases [2-5]. Although prospective data are still lacking, the number of PCs/EPCs is evidently inversely related to cardiovascular risk [6,7].

Since a clear and generally accepted definition of EPCs has until now not been established, most studies investigating the nature and function of EPCs have focused on flow-cytometric analysis of circulating cells that are positive for the haematopoietic stem cell markers CD34, CD133 and for the vascular endothelial growth factor receptor 2 (VEGFR2), and/or have concentrated on analysis of *in vitro *formation of colony-forming units (EPC-CFU) [1,6]. There is some evidence that levels of circulating progenitor cells positive for CD34 (CD34 + cells) are more strongly correlated with cardiovascular risk factors than are progenitor cell populations with various combinations of CD34, CD133, and VEGFR2 [6].

Women of reproductive age are exposed to lower cardiovascular risk than are age-matched men [8]. This is generally attributed to differences in sex hormones and, specifically, to the protective cardiovascular properties of female estrogens [9,10]. Notably, elevated estrogen plasma concentrations in women correlate with higher levels of circulating EPCs [11]. They accordingly promote vascular repair and inhibit neointima formation after carotid artery injury in mice [11]. The effects of estrogens on EPCs are mediated by nitric oxide and antiapoptotic mechanisms [11,12]. Changes in hormonal levels during the menstrual cycle are likewise reflected in the number of circulating progenitor cells. The highest levels of progenitor cells positive for CD34 and CD133 (CD34 + /CD133 +) were measured following the pre-ovulatory phase: i.e., the cycle phase with the highest levels of estrogens [13-15].

Smoking is the most important modifiable risk factor for atherosclerosis in young women [16]. The biological mechanisms linking smoking and atherogenesis are complex and not fully understood. In addition to inflammation, potential mechanisms by which smoking increases the risk of cardiovascular diseases include systemic haemostatic and coagulatory disturbances, lipid abnormalities, increase in oxidative stress, and vascular endothelial dysfunction [17-19]. It has been shown that smoking attenuates the number of progenitor cells in the circulatory system of male individuals, and that smoking cessation leads to a rapid elevation in progenitor cell levels [20]. It is presently unknown whether there is a link between smoking and the amount of progenitor cells in women in which endogenously released estrogens may further influence progenitor cell levels.

The aim of the present study was accordingly to investigate whether smoking influences the number of progenitor cells in young healthy women without other cardiovascular risk factors, and whether there is correlation between the amount of progenitor cells and endothelial function assessed as flow-mediated dilation (FMD) of the brachial artery.

## Methods

### Study population

Non-smoking and smoking women aged 25 to 35 years were recruited by press advertisements. Subjects with chronic diseases or known cardiovascular risk factors other than smoking were excluded. All women had regular menstrual cycles and had not taken medication for more than 3 months before study entry. The admitted non-smoking women had never smoked, and smoking women were required to have smoked at least 15 cigarettes/day for at least one year. Women were invited for preliminary examinations during the luteal phase at day M-7 of the previous menstrual cycle (day 1 is the first day of menstrual bleeding [M] of the expected following menstrual period). All values for clinical parameters were required to lie within the normal range to allow study inclusion: lipid profile including lipoprotein (a), blood pressure, body mass index, as well as routine internal and endocrine parameters. Serum progesterone was required to be > 18 nmol/L as a marker of an ovulatory menstrual cycle. The study protocol was approved by the Human Research Committee of the institution and conforms to the principles outlined in the Declaration of Helsinki. All subjects gave written informed consent.

### Study design

All volunteers underwent a clinical examination at the menstrual phase of the menstrual cycle (M + 3; day 3 after first day of menstrual bleeding). Blood from the antecubital vein was collected at 8.00 a.m. after a 12-hour overnight fast. The samples were centrifuged and stored at -70 °C until chemical analysis. For analysis of progenitor cells, blood was collected in EDTA vials and processed immediately. Endothelial function was measured by high-resolution vascular ultrasound. To assess the relevance of cyclical hormonal changes on progenitor cells, levels of these cells were additionally investigated in a subgroup of women at mid-follicular (M + 7; day 7 after first day of menstrual bleeding) and at mid-luteal (M-7) phases of the menstrual cycle. In this subgroup at M-7, the required minimum limit for progesterone was 18 nmol/L; otherwise the data were excluded from the analyses.

### EPC culture assay

Peripheral blood monocytic cells (PBMCs) were isolated from 18 ml of whole blood by density-gradient centrifugation with Ficoll-Paque™ (Amersham Pharmacia). Cells were plated in M199 medium (GIBCO 31153) with supplements (100 U/L penicillin, 100 μg/ml streptomycin, 20% fetal calf serum) on fibronectin-coated 24-well plates with a density of 1-10^6 ^cells per well. Nonadherent cells were removed after 3 days of culture, and colony forming units (CFU) were counted under light microscope on day 7 (6 wells per measuring point). Confirmation of endothelial like cell type was performed using the uptake of DiI-acetylated low density lipoprotein (acLDL-DiI) as indicator. Selected samples were positively tested for the expression of CD31 and endothelial nitric oxide synthase and for the formation of three dimensional tube structures in matrigel. Numbers of EPC were calculated as "EPC-CFU per 1 × 10^6 ^PBMCs".

### Flow cytometry

**Figure 1:**
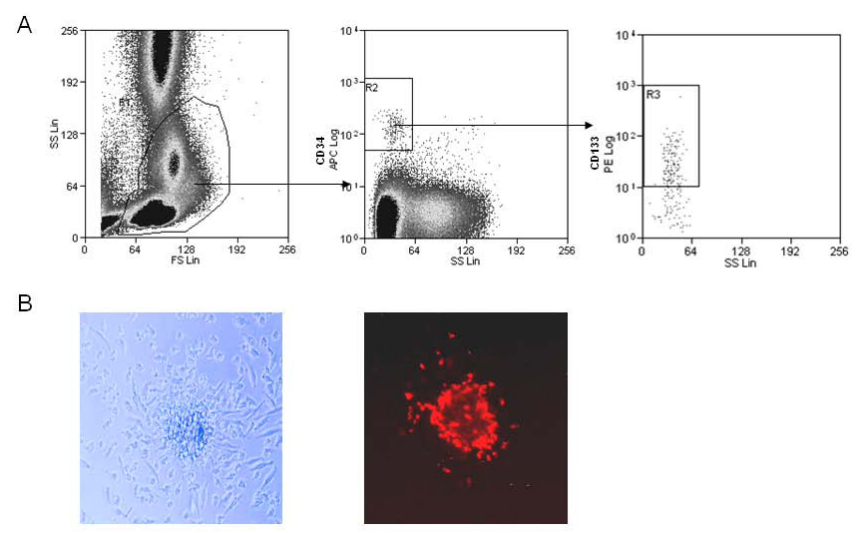
**Enumeration of circulating progenitor cells**. A) Detection of CD34 + side scatter^low ^and CD34 + /CD133 + side scatter^low ^cells, defining circulating progenitor cells, by flow cytometry. B) Endothelial progenitor cells analyzed by *in vitro *formation of colony forming unit (EPC-CFU) in a cell culture assay of peripheral blood mononuclear cells of young healthy women. Phase contrast photomicrograph of endothelial progenitor cell colony (EPC-CFU) with a central cluster of cells surrounded by migratory spindle shaped cells (left) and fluorescence photomicrograph after staining with acLDL-Dil (right).

Whole blood (0.5 ml) was stained with conjugated antibodies anti-CD34-APC (BD Pharmingen) and anti-CD133-PE (Miltenyi). Erythrocytes were subsequently lysed with EasyLyse Solution (Dako). Then the samples were washed with buffer containing phosphate-buffered saline and 0.5% bovine albumin. The numbers of circulating progenitor cells (CD34 + and CD34 + /CD133 +) were evaluated by flow cytometry (CyAn-ADP, BeckmanCoulter) as follows: The mononuclear cell fraction was identified by their low side scatter. Cells positive for CD34 were gated and examined for additional expression of CD133 (Figure 1A). Fluorescence-labeled, isotype-matched nonspecific immunoglobulin G antibodies served as controls for nonspecific staining. All instrument settings and the measurement procedure were stored in a protocol file and remained unchanged throughout the analyses. The gate on the mononuclear cell populations was set individually for each sample, and 5 × 10^5 ^PBMCs were measured. The concentrations of progenitor cells were calculated as "progenitor cells % PBMCs".

### Measurement of FMD and NMD

Endothelial function was measured by high-resolution vascular ultrasound with a 13-MHz linear array transducer (Sonoline Antares, Siemens), as recently described [21]. Briefly, endothelium-dependent FMD was assessed by measuring the change in brachial artery diameter after reactive hyperaemia for 2 minutes, according to established guidelines [22,23]. Endothelium-independent nitro-mediated dilation (NMD) was measured after sublingual application of nitro-glycerine spray (0.4 mg) for 6 minutes. FMD and NMD were defined as the maximum change in brachial artery diameter, in percent, compared to baseline measurement. Analyses of diameter changes were conducted offline (Tom Tec Imaging Systems) by two blinded investigators.

### Statistics

Values in tables and text are presented as mean ± standard deviation, and in the figures as mean ± SEM. All statistical tests were two-sided (level of significance = 0.05). Values with normal distribution were analysed by parametric tests. For comparisons of clinical parameters, which are not normally distributed, nonparametric rank-sum tests were used (paired Wilcoxon test, Mann-Whitney test). Correlations are given by the Spearman rank correlation coefficient. A linear regression analysis was performed to test the effect of adjusted age and smoking status on progenitor cells. The statistical model used for evaluation of the variables CD34 +, CD34 + /CD133 + and EPC-CFU over time (menstrual cycle) was a linear mixed model with fixed and random effects. The analyses were performed with Software SPSS version 16.0 and R version 2.6.2.

## Results

### Baseline characteristics

As described in Table 1, the study population consisted of 32 healthy women (mean age 31.3 ± 3.2 years) without overweight, hypertension, dyslipidemia including lipoprotein (a) diabetes, hyperhomocysteinemia, or family history of cardiovascular diseases. Nicotine abuse was the only cardiovascular risk factor established for 17 women within the study population. Smoking women were slightly older (32.4 ± 2.9 vs. 29.7 ± 2.9 years, p < 0.05). No other parameters showed significant differences between the two groups. Menstrual cycle was of normal length (mean cycle length 28.7 ± 2.1 days). Women of the subgroup that was additionally investigated at mid-follicular and luteal phases of the menstrual cycle (*n *= 20) showed the typical cyclical pattern in the concentrations of sexual steroids.

**Table 1:** Baseline characteristics of the study group (*n *= 32)

		Whole group *(n = 32)*	Non-smokers *(n = 15)*	Smokers *(n = 17)*
Age*	(years)	31.3 ± 3.2	29.7 ± 2.9	32.4 ± 2.9
BMI	(kg/m^2^)	21.3 ± 1.5	21.3 ± 1.7	21.3 ± 1.4
Cycle length	(days)	28.7 ± 2.1	29 ± 2.2	28.5 ± 1.5
Nicotine abuse	(pack years)	-	-	14.8 ± 4.6
RR syst	(mmHg)	98 ± 33	102 ± 30	94 ± 36
RR diast	(mmHg)	65 ± 22	67 ± 20	63 ± 25
Total cholesterol	(mg/dl)	164 ± 23	158 ± 17	168 ± 27
HDL	(mg/dl)	64.4 ± 10.1	66.9 ± 9.1	62.2 ± 10.7
LDL	(mg/dl)	85.8 ± 24.5	79.9 ± 16.5	91.1 ± 29.4
Triglyceride	(mg/dl)	67.7 ± 28.4	57.5 ± 13.4	76.7 ± 35.0
Lipoprotein(a)	(mg/dl)	7.0 ± 4.0	7.4 ± 4.2	6.6 ± 3.8
hsCrP	(mg/dl)	0.08 ± 0.07	0.09 ± 0.08	0.08 ± 0.06
Insulin	(mIU/l)	5.6 ± 2.2	5.2 ± 2.1	6.0 ± 2.3
Glucose	(mg/dl)	87.7 ± 7.2	87.0 ± 4.9	88.4 ± 8.8
Homocystein	(μmol/l)	8.5 ± 1.7	8.2 ± 1.7	8.7 ± 1.8
Folic acid	(μg/l)	8.8 ± 4.5	10.2 ± 5.6	7.6 ± 2.8
Uric acid	(μmol/l)	3.9 ± 0.9	3.7 ± 0.9	4.1 ± 0.8
Estradiol	(pmol/l)	613 ± 194	598 ± 191	626 ± 201
Progesterone	(nmol/l)	51.4 ± 17.6	51.2 ± 16.4	51.5 ± 19.2
LH	(IU/l)	4.7 ± 2.9	4.5 ± 3.8	4.9 ± 2.0
FSH	(IU/l)	2.7 ± 1.2	2.8 ± 1.5	2.7 ± 0.7

*p < 0.05 for comparison of non-smokers with smokers; values are mean ± SD;

BMI = body mass index; LH = luteinising hormone; FSH = follicle stimulating hormone; RR syst = systolic blood pressure; RR diast = diastolic blood pressure; HDL = high-density lipoprotein; LDL = low-density lipoprotein; hsCrP = high-sensitivity C-reactive protein.

### Effect of smoking on the number of circulating progenitor cells in young healthy women

At menstrual phase (M + 3; day 3 after first day of menstrual bleeding) in the 17 smoking women, we found an abundance of CD34 + cells that was significantly lower than among the 15 non-smoking women (0.06 ± 0.02 vs. 0.10 ± 0.03% of PMBC, p = 0.01). The CD34 + /CD133 + progenitor cell number was also lower in smoking women, close to significance (p = 0.056). The numbers are in the expected range when compared to studies using similar evaluation methods [24,25].

**Figure 2:**
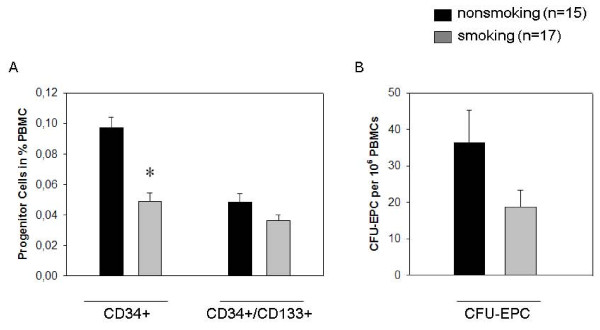
**Influence of smoking on the number of circulating progenitor cells**. A) CD34 + and CD34 + /CD133 + cell populations in peripheral blood measured by flow cytometry. Level of circulating CD34 + progenitor cells is significantly decreased by smoking in young healthy women. B) Endothelial progenitor cells analyzed by *in vitro *formation of colony forming unit (EPC-CFU) in a cell culture assay of peripheral blood mononuclear cells. Number of EPC-CFU is decreased by smoking in young healthy women. *p < 0.05 for comparison of non-smokers with smokers; values are mean ± SEM.

The mean value of EPC-CFU was clearly lower for smoking than for non-smoking women (18.8 ± 18.6 vs. 36.5 ± 34.0 per 10^6 ^PBMC). However, due to extensive variations within the groups, the difference is not statistically significant (Figure 2). In a similar manner, FMD was slightly but not significantly reduced in smoking women. NMD did not show significant differences in the two groups. All results are presented in detail in Table 2.

**Table 2:** Summary of results for M + 3

		Whole group *(n = 32)*	Non-smokers *(n = 15)*	Smokers *(n = 17)*
CD34+	(% of PMBC)	0.08 ± 0.03	0.10 ± 0.03	0.06 ± 0.02*
CD34+/CD133+	(% of PMBC)	0.04 ± 0.02	0.05 ± 0.02	0.04 ± 0.01
EPC-CFU	(per 10^6 ^PBMC)	27.1 ± 28.0	36.5 ± 34.0	18.8 ± 18.6
FMD	(%)	9.2 ± 4.1	9.9 ± 4.5%	8.5 ± 3.7
NMD	(%)	25.0 ± 7.3	24.3 ± 7.2	25.7 ± 7.5
Estradiol	(pmol/l)	151 ± 75	175 ± 76	130 ± 70
Progesterone	(nmol/l)	3.86 ± 1.54	3.12 ± 1.29	4.5 ± 1.48

*p < 0.05 for comparison of non-smokers with smokers; values are mean ± SD; CD34+ = progenitor cells positive for haematopoietic stem cell marker CD34; CD34+/CD133+ = progenitor cells positive for hematopoietic stem cell markers CD34 and CD133; EPC-CFU = endothelial progenitor cells analyzed by in vitro formation of colony forming unit; FMD = flow-mediated dilation; NMD = nitro-mediated dilation

We next analyzed the number of progenitor cells in the subgroup of women investigated at luteal and follicular phases of the menstrual cycle. Results are summarized in Table 3. Only women with an ovulatory menstrual cycle were included in this subgroup (n = 20). Concentration of sexual steroids showed the typical cyclical pattern, with significantly higher levels of estradiol and progesterone at mid-luteal phase than in the menstrual and mid-follicular phases (8 non-smoking women, 12 smoking women). Neither the number of CD34 + and CD34 + /CD133 + progenitor cells nor the number of EPC-CFU showed significant differences between the three investigated time points of the menstrual cycle. However, in correlation with results found at the menstrual phase, CD34 + cell levels were significantly lower in smoking than in non-smoking women at mid-follicular and mid-luteal phases. The numbers of EPC-CFU showed a non-significant trend towards lower values in smoking women, whereas CD34 + /CD133 + progenitor cell counts were not influenced by smoking in this subgroup (Table 3).

**Table 3:** Levels of progenitor cells during the menstrual cycle

		non-smokers *(n = 8)*			Smokers *(n = 12)*		
		**M + 3**	**M + 7**	**M - 7**	**M + 3**	**M + 7**	**M - 7**

CD34+	(%PMBC)	0.08 ± 0.03	0.09 ± 0.03	0.08 ± 0.02	0.06 ± 0.02*	0.06 ± 0.02*	0.06 ± 0.01*
CD34+/CD133+	(%PMBC)	0.04 ± 0.02	0.04 ± 0.02	0.04 ± 0.02	0.04 ± 0.01	0.02 ± 0.01	0.03 ± 0.01
EPC-CFU	(per10^6 ^PBMC)	45.8 ± 12.5	49.3 ± 22	28.2 ± 6.4	21.9 ± 18.8	14.5 ± 21.3	16.4 ± 19.1
estradiol	(pmol/l)	188 ± 79	353 ± 119	664 ± 138	138 ± 78	285 ± 150	545 ± 128
progesterone	(nmol/l)	2.6 ± 0.6	1.7 ± 0.4	55.3 ± 26.9	4.8 ± 1.6	2.8 ± 0.8	47.1 ± 16.8

*p < 0.05 for comparison of non-smokers vs. smokers; values are mean ± SD; CD34+ = progenitor cells positive for hematopoietic stem cell marker CD34; CD34+/CD133+ = progenitor cells positive for hematopoietic stem cell markers CD34 and CD133; EPC-CFU = endothelial progenitor cells analyzed *in vitro *by formation of colony forming units

### Number of circulating CD34 + cells in young healthy women correlates with endothelial function

**Figure 3:**
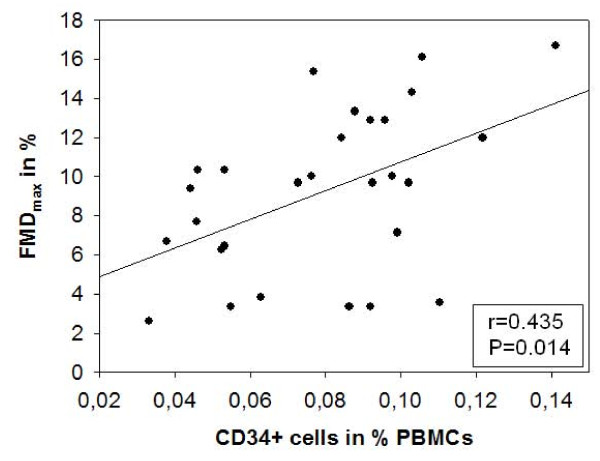
**Relation between the number of circulating CD34 + progenitor cells and endothelial function**. Measured as flow-mediated dilation (FMD) in the brachial artery in young healthy women.

We examined women at menstruation, the cycle phase with lowest concentrations of sexual steroids. Levels of EPC-CFU showed broad interindividual distribution (0.7 to 118.3 cells per 10^6 ^per PBMCs). Levels of circulating progenitor cells estimated by flow cytometry ranged from 0.03% to 0.145% and 0.02% to 0.10% of PBMCs for CD34 + and CD34 + /CD133 + progenitor cells, respectively. The number of CD34 + progenitor cells revealed a significant positive correlation with FMD (r = 0.435, p = 0.014) (Figure 3). Levels of CD34 + /CD133 + progenitor cells and of EPC-CFU, however, showed no correlation with FMD. NMD likewise failed to correlate with the number of CD34 + cells, CD34 + /CD133 + cells, or EPC-CFU. Adjustment of FMD, number of CD 34 + cells, and number of CD34 + /CD133 + cells for age did not disclose an influence of this variable.

## Discussion

The major observation of the present study is that levels of circulating progenitor cells and endothelial progenitor cells are decreased in healthy young women with chronic nicotine abuse. In addition, the number of CD34 + progenitor cells positively correlates with endothelial function assessed as flow-mediated dilation of the brachial artery.

Previous studies have shown that chronic nicotine abuse leads to a decrease in circulating progenitor cell levels in men [20]. Two hypotheses have been discussed to explain underlying mechanisms: First, smoking-related ROS production may decrease the bioavailability of nitric oxide (NO), thereby reducing mobilization of PCs from the bone marrow [26,27]. Second, smoking leads to endothelial dysfunction, and injured vessels may use PCs to maintain endothelial function [20]. An imbalance of injury and repair of vasculature promotes the progression of atherosclerosis, and reduced PC levels may be a marker of such an imbalance caused by smoking [27].

PCs are known to be upregulated by estrogens through inhibition of apoptosis, stimulation of telomerase, and bone marrow mobilization [11,28,29]. Fertile women have higher levels of PCs than do age-matched men; after menopause, levels of PCs no longer evidence sex-specific differences [14]. Higher PC levels in fertile women have been suggested to contribute to the lower cardiovascular risk compared to men. In order to explain the effect of smoking and the estrogen status on progenitor cell and EPC-CFU content, we first examined women at menstruation, the cycle phase with lowest concentrations of sexual steroids. We established a significant decrease of CD34 + progenitor cells in smoking women. Importantly - in a cross-analysis of six different progenitor cell subtypes characterized by different combinations of the surface markers CD34, CD133, and VEGFR2 - it had been previously shown that the abundance of the progenitor cell population with CD34 + alone revealed the best correlation with cardiovascular parameters and risk estimates [6]. Concordantly, we found a positive correlation of levels of CD34 + cells with endothelial function in young healthy women. These data concur with other studies describing a positive correlation between FMD and PCs: which emphasizes the close relationship of these two markers in reflection of vascular homeostasis [6,14].

It is well established that cigarette smoking is sufficient to impair endothelial function in healthy adults [18,30]. Although hormonal cycle-dependent changes of endothelial function are relevant in premenopausal women, most clinical studies of the effects of smoking on endothelial-dependent vasodilation have not considered menstrual cycle phases in women. We have recently shown that there are no significant differences in endothelial function between smoking and non-smoking women at menstruation [31]. Consequently, the results of the present study support the conclusion that chronic smoking affects the level of CD34 + progenitor cells before manifestation of endothelial dysfunction in healthy young women. It has been suggested that oxidative stress and decreased availability of NO are causative for endothelial dysfunction induced by smoking [32,33]. Since there is evidence that NO is necessary for PC mobilization and function [26,27,34], NO deficiency may be a key factor resulting in the PC decrease in young smoking women. We suggest, that deficient mobilization of PCs due to limited NO availability caused by smoking precedes detectable alterations of endothelial function and in this sense may represent a sensitive, early parameter of cardiovascular risk. Clearly, this should be an important consideration for future studies.

Investigations of the influence of nicotine abuse on PC levels in premenopausal women should in fact consider hormonal fluctuations during the menstrual cycle. In our study, however, we found no significant differences in the amount of progenitor cells between follicular and luteal phases of the menstrual cycle, despite an increase in estrogen levels. These results concur with previous studies showing significant periovulatory increase of progenitor cells, but no differences between follicular and luteal phases [13-15]. It is interesting to note that the differences in levels of CD34 + cells between smoking and non-smoking women were maintained in all three phases, independently of the estrogen status.

There are some limitations in the present study. In conjunction with the small sample size, smoking women were somewhat older than non-smoking. It cannot be ruled out that older age in smoking women contributes to decreased numbers of progenitor cells. However, adjustment of progenitor cell number and FMD values in a linear regression analysis did not show an influence of age differences on our study results.

## Conclusion

The number of CD34 + progenitor cells positively correlates with FMD in young healthy women and is decreased by smoking.

## Competing interests

The authors declare that they have no competing interests.

## Authors' contributions

AL and NJ contributed to the concept and design of the study, data collection, analysis and interpretation of data, and drafting of the manuscript. AK, CK, SM contributed to data collection and analysis. CG performed the statistical analysis. GB, KS, VS contributed to the concept and design of the study, and critical review of the intellectual content of the manuscript. All authors read and approved the final manuscript.

## Pre-publication history

The pre-publication history for this paper can be accessed here:

http://www.biomedcentral.com/1472-6874/10/20/prepub
